# The Safety and Tolerability of Linezolid in Novel Short-Course Regimens Containing Bedaquiline, Pretomanid, and Linezolid to Treat Rifampicin-Resistant Tuberculosis: An Individual Patient Data Meta-analysis

**DOI:** 10.1093/cid/ciad653

**Published:** 2023-10-24

**Authors:** Tasnim Hasan, Ellie Medcalf, Bern-Thomas Nyang'wa, Erica Egizi, Catherine Berry, Matthew Dodd, Salah Foraida, Medea Gegia, Mengchun Li, Fuad Mirzayev, Hannah Morgan, Ilaria Motta, Linh Nguyen, Samuel Schumacher, Tim Schlub, Greg Fox

**Affiliations:** Faculty of Medicine and Health, University of Sydney, Sydney, Australia; Faculty of Medicine and Health, University of Sydney, Sydney, Australia; Public Health Department, Médecins sans Frontières, Amsterdam, The Netherlands; Clinical Research Department, London School of Hygiene and Tropical Medicine, London, United Kingdom; TB Alliance Research and Development, New York City, USA; Manson Unit, Médecins sans Frontières, London, United Kingdom; Department of Medical Statistics, London School of Hygiene and Tropical Medicine, London, United Kingdom; TB Alliance Research and Development, New York City, USA; Global Tuberculosis Program, World Health Organization, Geneva, Switzerland; TB Alliance Research and Development, New York City, USA; Global Tuberculosis Program, World Health Organization, Geneva, Switzerland; Faculty of Medicine and Health, University of Sydney, Sydney, Australia; Manson Unit, Médecins sans Frontières, London, United Kingdom; Global Tuberculosis Program, World Health Organization, Geneva, Switzerland; Global Tuberculosis Program, World Health Organization, Geneva, Switzerland; Faculty of Medicine and Health, University of Sydney, Sydney, Australia; Faculty of Medicine and Health, University of Sydney, Sydney, Australia

**Keywords:** multidrug-resistant tuberculosis, BPaL, linezolid, severe adverse events

## Abstract

**Background:**

Effectiveness, safety, tolerability, and adherence are critical considerations in shifting to shorter tuberculosis (TB) regimens. Novel 6-month oral regimens that include bedaquiline (B), pretomanid (Pa), and linezolid (L), with or without a fourth drug, have been shown to be as or more effective than the established longer regimens for the treatment of multidrug-resistant/rifampicin-resistant TB (MDR/RR-TB). We aimed to evaluate the safety and tolerability of linezolid in BPaL-containing regimens for the treatment of MDR/RR-TB among recently completed clinical trials.

**Methods:**

A review and meta-analysis was undertaken including published and unpublished data from clinical trials, conducted between 2010 and 2021, that evaluated regimens containing BPaL for the treatment of MDR/RR-TB. Individual patient data were obtained. For each BPaL-containing regimen, we evaluated the frequency and severity of treatment-related adverse events. The risk difference of adverse events for each regimen was calculated, in comparison to patients assigned to receiving the lowest cumulative exposure of linezolid.

**Results:**

Data from 3 clinical trials investigating 8 unique BPaL-containing regimens were included, comprising a total of 591 participants. Adverse events were more frequent in groups randomized to a higher cumulative linezolid dose. Among patients who were randomized to a daily dose of 1200 mg linezolid, 68 of 195 (35%) experienced a grade 3–4 adverse event versus 89 of 396 (22%) patients receiving BPaL-containing regimens containing 600 mg linezolid.

**Conclusions:**

Regimens containing BPaL were relatively well tolerated when they included a daily linezolid dose of 600 mg. These novel regimens promise to improve the tolerability of treatment for MDR/RR-TB.

## Teaser

Different bedaquiline, pretomanid, and linezolid–containing regimens from recently published clinical trials for the treatment of drug-resistant tuberculosis are compared. Different linezolid doses and durations were used in these regimens, allowing for an assessment of the safety of linezolid.

Every year there are approximately half a million new cases of multidrug-resistant/rifampicin-resistant tuberculosis (MDR/RR-TB) diagnosed globally, with 150 000 deaths [1]. Over the last decade, the treatment success rate for patients with MDR/RR-TB has increased from 50% to 60% [1]. New and repurposed antibiotics promise to shorten the duration, improve the tolerability, and improve the efficacy of treatment for MDR/RR-TB: *Mycobacterium tuberculosis* that is resistant to the 2 most effective, first-line anti-TB antibiotics, isoniazid and rifampicin [2, 3].

Between 2019 and 2022, the World Health Organization (WHO)–recommended regimens, based on data from programmatic settings, for the treatment of MDR/RR-TB included a minimum of 5 drugs. These included the 3 most effective “group A drugs” (bedaquiline, linezolid, and a fluoroquinolone), where tolerated and susceptible [2]. The treatment duration recommended in these guidelines varied from a 9-month (“short course”) regimen, for selected patients to a longer 18-month regimen. However, toxicity commonly associated with these regimens included hepatotoxicity and hematological and neurological toxicity due to the use of injectable agents and prolonged therapy [4].

In 2022, WHO commissioned a revision to the MDR/RR-TB treatment guidelines, following the completion of clinical trials that evaluated all-oral regimens including the antibiotics bedaquiline (B), pretomanid (Pa), and linezolid (L) [5–7]. The TB pragmatic clinical trial for a more effective, concise and less toxic regimen (PRACTECAL), Nix, and ZeNix trials evaluated treatment outcomes of a number of BPaL regimens, with varying linezolid doses [5, 6]. The safety of regimens containing BPaL, with or without the addition of a fourth antimicrobial agent (henceforth, called BPaL-containing regimens), remains an important consideration. Linezolid is an oxazolidinone antibiotic that has been repurposed for use in treatment of TB. Particularly at high doses, linezolid has been associated with peripheral neuropathy, optic neuropathy, and bone marrow toxicity [6, 8]. In contrast, bedaquiline, a diarylquinoline that blocks ATP synthase, and pretomanid, a nitroimidazole, are generally well tolerated. However, QT prolongation and hepatotoxicity have been reported [9, 10].

We undertook a review and individual patient data meta-analysis as a part of the 2022 WHO Guideline Development Group process. The study aimed to compare the cumulative incidence of adverse events among patients taking linezolid in 6-to-9-month BPaL-containing regimens for the treatment of MDR/RR-TB within recent clinical trials.

## METHODS

### Study Design and Participants

A review and individual patient data analysis were undertaken, and reported using the PRISMA (Preferred Reporting Items for Systematic Reviews and Meta-Analyses) guidelines [11]. We included clinical trials for which results were available on 30 September 2021. Studies included patients with MDR/RR-TB with additional resistance to a fluoroquinolone antibiotic or second-line injectable agent (pre-extensively drug resistant [preXDR]-TB) or extensively drug resistant (XDR)-TB (resistance to both fluoroquinolone and second-line injectable agents), who were treated with BPaL-containing regimens. We used the 2018 WHO definitions for XDR-TB [12].

### Search Strategy

A public call was issued in July 2021 by the WHO Global TB Program (GTP) to identify studies that met the following criteria: (1) parallel-group or single-arm clinical trials, who were treated with BPaL, with or without an additional “companion” drug, regardless of dose and duration of the regimen; (2) including patients with bacteriologically confirmed MDR/RR-TB that was either pulmonary or extra-pulmonary; (3) including at least 25 patients commencing treatment; and (4) availability of individual participant data, including the individual regimen(s) used, the duration of treatment, and for which sufficient data were available to allow assignment of treatment outcomes for the majority of participants. Datasets were excluded if they were not a clinical trial and if they did not investigate a BPaL-containing regimen. Consultation was done with experts in the field and by searching public clinical trial registries to identify trials not yet published. A search of databases (MEDLINE, PubMed, EMBASE) did not reveal further studies that met the inclusion criteria.

### Data Collection

Contributors were asked to provide complete datasets and study protocol with de-identified individual patient data, which were reviewed for safety and efficacy outcomes. Participants were included in the final analysis for both the standard-of-care and BPaL-containing regimen, if all of the following criteria were met: (1) bacteriologically confirmed *M. tuberculosis*; (2) rifampicin resistance or MDR/RR-TB, confirmed by genotypical or phenotypical drug susceptibility testing; (3) any age; (4) pulmonary or extra-pulmonary TB; (5) treatment outcomes that could be classified according to WHO definitions [3]; and (6) a defined treatment regimen including information about composition and treatment duration. Participants within a trial were excluded from the present analysis if the patient received treatment exceeding 12 months in duration.

Adverse events were classified according to each individual trial based on the Common Terminology Criteria for Adverse Events version 5 (grade 1 to 4) [13] or the Division of Microbiology and Infectious Diseases [14] Adult Toxicity Table 2007 (draft) (grade 1 to 4; Supplementary Table 1). Adverse events of special interest (AESI) to the WHO GTP were specified a priori based upon common and serious toxicities known to occur with bedaquiline, pretomanid, and linezolid. These included bone marrow toxicity, peripheral neuropathy (henceforth, defined collectively by terms using the Standardized MedDRA Queries [SMQ] [15]), optic neuropathy, QT interval (QTc) prolongation, and hepatotoxicity. A severe adverse event was defined as being an adverse event of grade 3–4. In the primary intention-to-treat population, participants were classified according to their intended dose and duration of linezolid at the time of randomization.

End-of-treatment outcomes were reported for each regimen according to the 2020 WHO MDR/RR-TB outcome definitions [3]. Successful treatment outcomes encompassed those who achieved an outcome of cure or treatment completed. Unfavorable treatment outcomes comprised individuals with an outcome of failed treatment or who died or were lost to follow-up [3].

The intended treatment duration was defined as the duration assigned to each participant at the time of commencing MDR/RR-TB treatment, according to the study protocol. The intended treatment dose for linezolid was defined as the dose of linezolid assigned to each participant at the time of treatment commencement (1200 mg or 600 mg). The actual treatment duration was calculated as the number of weeks for which the treatment regimen or drug was actually used, excluding periods of drug interruption. Treatment discontinuation occurred when 1 or more drugs within the intended regimen was permanently stopped, without recommencement of the same drug or regimen.

### Statistical Analysis

Descriptive statistics were used to describe participants’ demographic and clinical features. Comparisons were made between arms of each trial; however, similar regimens (eg, regimens using the exact same drugs) between trials were not combined due to differences in the doses, adverse event monitoring, and setting between trials. The cumulative incidence was calculated for all adverse events, AESI resulting in treatment discontinuation, and for all grade 3–4 AESI. Adverse events within each study arm were compared with a reference group, which was identified as the group with the lowest intended linezolid exposure. Differences in the proportion of adverse events are presented as a risk difference (RD) with 95% confidence intervals (CIs), calculated using the Score method. Statistical analyses were performed using SAS version 9.4 (SAS Institute) and RStudio 2022.02.2 + 485 (R Foundation for Statistical Computing).

### Ethical Issues

Ethical approval for the included trials was provided by the institutional review boards of each responsible ethics committee (TB Alliance and Médecins sans Frontières). Approval to share data for this study was provided by the trial steering committees or sponsor. No additional data were obtained. This work was funded by the World Health Organisation.

## RESULTS

Only 3 trials were identified, and all met the eligibility criteria. A total of 8 unique BPaL-containing regimens were included (Table 1 and Supplementary Table 1). In the Nix-TB trial participants were assigned to BPaL with 1200 mg of linezolid for up to 26 weeks (Nix-TB 1200-26) [5]. In the ZeNix trial participants were randomized to one of four 26–39-week treatment groups with (1) linezolid 600 mg for 9 weeks (ZeNix 600-9), (2) linezolid 600 mg for 26 weeks (ZeNix 600-26), (3) linezolid 1200 mg for 9 weeks (ZeNix 1200-9), or (4) linezolid 1200 mg for 26 weeks (ZeNix 1200-26) [6]. The primary outcome for both the Nix-TB and ZeNix trials was the incidence of unfavorable outcomes defined as treatment failure or relapse of disease [5, 6].

**Table 1.** Characteristics of the Nix, ZeNix, and TB-PRACTECAL Trials

Trial Name	Nix-TB Trial	ZeNix Trial	TB-PRACTECAL Trial
Study design	Single-arm intervention trial	Randomized controlled trial	Phase 2/3 randomized controlled trial
Blinding	Unblinded	Partially blinded (participants and investigators were blinded to linezolid dose and duration)	Open-blinded
Study population	Participants with XDR-TB (2018 definition) or treatment-intolerant nonresponsive MDR/RR-TB	Participants with XDR-TB (2018 definition) or treatment-intolerant nonresponsive MDR-TB	Microbiologically confirmed *Mycobacterium tuberculosis* with resistance to rifampicin
Number of participants	108	172	419
Investigational arm(s)	Bedaquiline, pretomanid, linezolid daily orally	Arm 1: bedaquiline, pretomanid with linezolid 1200 mg daily for 26 wk orally Arm 2: bedaquiline, pretomanid, with linezolid 1200 mg daily for 9 wk orally Arm 3: bedaquiline, pretomanid, with linezolid 600 mg daily for 26 wk orally Arm 4: bedaquiline, pretomanid, with linezolid 600 mg daily for 9 wk orally	Arm 1: bedaquiline, pretomanid, linezolid, moxifloxacin (BPaLM) daily orally Arm 2: bedaquiline, pretomanid, linezolid, clofazamine (BPaLC) daily orally Arm 3: Bedaquiline, pretomanid, linezolid (BPaL) daily orally
Standard-of-care regimen	None	None	Local standard-of-care regimen^a^
Duration of intervention regimen	26 wk	26 wk	24 wk
Linezolid dose	1200 mg daily or 600 mg twice daily	Arm 1: 1200 mg daily for 26 wk Arm 2: 1200 mg daily for 9 wk Arm 3: 600 mg daily for 26 wk Arm 4: 600 mg daily for 9 wk	All intervention arms 600 mg daily for 16 wk, followed by daily 300 mg for 8 wk
Modifications allowed to linezolid	Linezolid dose could be reduced, interrupted or permanently discontinued if linezolid-related toxicity was suspected.	Linezolid dose could be reduced, interrupted, or permanently discontinued if linezolid-related toxicity was suspected.	Linezolid dose could be reduced or interrupted for up to 2 wk; however, discontinuation of linezolid resulted in discontinuation of the participant from the trial.
Ratio of randomization	Not applicable	1 : 1:1 : 1	1 : 1:1 : 1
International sponsor	TB Alliance	TB Alliance	Médecins sans Frontières
Inclusion criteria	≥14 y age Pulmonary TB Fluoroquinolone or injectable resistance Intolerance to previous MDR/RR-TB treatment	≥14 y age Pulmonary TB Fluoroquinolone or injectable resistance Intolerance to previous MDR-TB treatment	≥15 y Microbiologically confirmed rifampicin resistance Pulmonary and extra-pulmonary TB
Exclusion criteria	BMI <17 kg/m^2^ Prolonged QTc >500 ms Heart failure Pregnancy Suspected resistance to B, Pa, L CD4 <100 cells/mm^3^ Those who were moribund	BMI <17 kg/m^2^ Prolonged QTc >500 ms Heart failure Pregnancy Suspected resistance to B, Pa, L CD4 <100 cells/mm^3^ Those who were moribund	Prolonged QTc >450 ms Hepatitis History of cardiac disease Pregnancy Suspected resistance to B, Pa, L Those who were moribund
Primary endpoint	Incidence of bacteriological failure or relapse at 12 mo post–treatment initiation	Incidence of bacteriological failure or relapse at 12 mo post–treatment initiation	Unfavorable outcome at 72 wk post-randomization (composite of death, treatment failure, treatment discontinuation, loss to follow-up, recurrence, still on treatment at 72 wk)
Adverse event classification	Severity (grades 1 to 4) Serious adverse events (death, life-threatening, prolonged hospitalization, persistent disability, birth defect, other serious event)	Grades 1 to 4 Serious adverse events (death, life-threatening, prolonged hospitalization, persistent disability, birth defect, other serious event)	Grades 1 to 4 Serious adverse events (death, life-threatening, prolonged hospitalization, persistent disability, birth defect, other serious event)
Drug susceptibility testing	MGIT drug-susceptibility testing	MGIT drug-susceptibility testing	MGIT drug-susceptibility testing
Follow-up duration	104 wk post–treatment completion	78 wk post–treatment completion	108 wk post–treatment initiation
Location	South Africa	South Africa, Russia, Georgia, Moldova	South Africa, Uzbekistan, Belarus
Healthcare settings	Three hospitals in South Africa, including inpatients and outpatients	Eleven dedicated trial centers, including inpatients and outpatients	Uzbekistan: hospitals including outpatient clinics and TB treatment centers South Africa: 4 hospitals including inpatients and outpatients Belarus: patients initially hospitalized

The monitoring of adverse events is summarized in Supplementary Table 1.

Abbreviations: B, bedaquiline; BMI, body mass index; L, linezolid; MDR/RR, multidrug-resistant/rifampicin-resistant; MGIT, Mycobacteria growth indicator tube; Pa, pretomanid; QTc, QT interval; TB, tuberculosis; TB PRACTECAL, Pragmatic Clinical Trial for a more Effective, Concise and Less Toxic Regimen, XDR, extensively drug resistant.

^a^The standard-of-care regimens were a composite of acceptable regimens at the study sites at the time of enrollment. These include the standard short-course regimen (9 months injectable-based ¨Bangladesh regimen¨, all oral bedaquiline-based short regimens, injectable-based long-course regimen, all oral bedaquiline-based long-course regimens).

The TB-PRACTECAL trial aimed to evaluate the safety and efficacy of 3 intervention regimens against a composite of locally accepted standards of care (including regimens with injectables and regimens of 18–24 months in duration) [7]. The 3 intervention regimens were 24 weeks of BPaL, BPaL with moxifloxacin (BPaLM), or BPaL with clofazimine (BPaLC). The dose of linezolid in each arm was 600 mg daily for 16 weeks, then 300 mg daily for the remaining 8 weeks, or earlier if not well tolerated. Permanent cessation of linezolid alone was not permitted in TB-PRACTECAL. Enrollment in the TB-PRACTECAL trial was terminated in March 2021 on the recommendation of the Data and Safety Monitoring Board, after an interim analysis demonstrated superior efficacy for the BPaLM investigational arm in comparison to the standard of care [7].

A total of 591 participants who were assigned BPaL or a BPaL-containing regimen were included from the 3 trials. Furthermore, an additional 108 participants were assigned to a standard-of-care regimen in the TB-PRACTECAL study. Demographic characteristics of the participants by regimen are shown in Table 2. A higher proportion of participants were resistant to fluoroquinolones in the Nix-TB and ZeNix trials (60–76%) compared with TB-PRACTECAL (19–26%). The Nix trial had the highest proportion of participants who were persons with human immunodeficiency virus (HIV) (51%). Successful end-of-treatment outcomes were reported for more than 80% of participants in all trial arms (Supplementary Table 2).

**Table 2.** Characteristics of the Study Populations, Stratified by Regimen

Clinical characteristics	Nix-TB Trial	ZeNix Trial				TB-PRACTECAL Trial			
	BPaL 1200-26 Nix-TB	BPaL 600-9 ZeNix	BPaL 600-26 ZeNix	BPaL 1200-9 ZeNix	BPaL 1200-26 ZeNix	BPaL TB-PRACTECAL	BPaLM TB-PRACTECAL	BPaLC TB-PRACTECAL	SoC TB-PRACTECAL
Total	108	42	43	43	44	105	104	102	108
Age, mean (SD), y	35.5 (10.1)	37.6 (9.8)	34.8 (11.1)	39.1 (11.6)	36.4 (9.1)	37.2 (11.5)	36.5 (11.5)	33.6 (10.1)	38.3 (10.7)
Adults aged ≥18 y	106 (98%)	42 (100%)	43 (100%)	43 (100%)	44 (100%)	104 (99%)	104 (100%)	101 (99%)	108 (100%)
Male	57 (53%)	29 (69%)	29 (67%)	27 (63%)	15 (34%)	60 (57%)	67 (64%)	53 (52%)	63 (58%)
Country									
South Africa	108 (100%)	15 (36%)	20 (47%)	18 (42%)	10 (23%)	35 (33%)	36 (35%)	35 (34%)	37 (34%)
Georgia	0 (0%)	8 (19%)	4 (9%)	8 (19%)	13 (30%)	0 (0%)	0 (0%)	0 (0%)	0 (0%)
Russia	0 (0%)	16 (38%)	18 (42%)	13 (30%)	19 (43%)	0 (0%)	0 (0%)	0 (0%)	0 (0%)
Moldova	0 (0%)	3 (7%)	1 (2%)	4 (9%)	2 (5%)	0 (0%)	0 (0%)	0 (0%)	0 (0%)
Uzbekistan	0 (0%)	0 (0%)	0 (0%)	0 (0%)	0 (0%)	50 (48%)	50 (48%)	48 (47%)	51 (47%)
Belarus	0 (0%)	0 (0%)	0 (0%)	0 (0%)	0 (0%)	20 (19%)	18 (17%)	19 (19%)	20 (19%)
HIV positive	55 (51%)	9 (21%)	9 (21%)	8 (19%)	8 (18%)	27 (26%)	24 (23%)	33 (32%)	28 (26%)
Past TB^a^	90 (83%)	30 (71%)	30 (70%)	31 (72%)	28 (64%)	43 (41%)	42 (40%)	47 (46%)	47 (44%)
Past drug-resistant TB^b^	0 (0%)	0 (0%)	0 (0%)	0 (0%)	0 (0%)	17 (16%)	11 (11%)	14 (14%)	10 (9%)
Smear positive at diagnosis	78 (72%)	35 (83%)	34 (79%)	31 (72%)	39 (89%)	71 (68%)	72 (69%)	69 (68%)	74 (69%)
Culture positive at diagnosis^c^	102 (94%)	34 (81%)	32 (74%)	30 (70%)	35 (80%)	95 (90%)	93 (89%)	86 (84%)	96 (89%)
Fluoroquinolone-resistant at diagnosis^d^	82 (76%)	27 (64%)	33 (77%)	26 (60%)	31 (70%)	27 (26%)	20 (19%)	25 (25%)	27 (25%)

Data are presented as n (%) unless otherwise indicated.

Abbreviations: BPaLM, bedaquiline, pretomanid, linezolid with moxifloxacin; HIV, human immunodeficiency virus; MDR/RR-TB, multidrug-resistant/rifampicin-resistant TB; SD, standard devation; SoC, standard of care; TB, tuberculosis; TB PRACTECAL Pragmatic Clinical Trial for a more Effective, Concise and Less Toxic Regimen.

^a^Previous treatment episode for TB regardless of susceptibility.

^b^Previous treatment episode for MDR/RR-TB.

^c^Defined as a positive sputum culture for TB prior to the initiation of treatment.

^d^Individuals with fluoroquinolone resistance in the BPaLM regimen received moxifloxacin.

**Figure 1.**
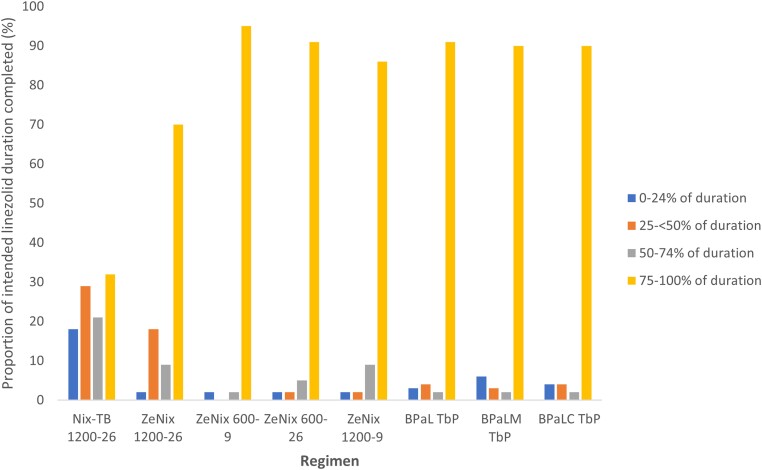
Proportion of individuals receiving each BPaL-containing regimen who completed the intended duration of linezolid treatment. For ZeNix 600-9 the intended linezolid dose and duration was 600 mg daily for 9 weeks, for ZeNix 600-26 the intended linezolid dose and duration was 600 mg daily for 26 weeks, for ZeNix 1200-9 the intended linezolid dose and duration was 1200 mg daily for 9 weeks, for ZeNix 1200-26 the intended linezolid dose and duration was 1200 mg daily for 26 weeks, and for Nix-TB the intended linezolid dose and duration was 600 mg daily for 26 weeks. For all TbP the intended linezolid dose and duration was 600 mg daily for 16 weeks and then 300 mg daily for 9 weeks, or earlier if moderately tolerated. Abbreviations: BPaL, bedaquiline, pretomanid, and linezolid; BPaLC, bedaquiline, pretomanid, and linezolid with clofazimine; BPaLM, bedaquiline, pretomanid, and linezolid with moxifloxacin; TbP, TB PRACTECAL.

Figure 1 presents the proportion of the intended linezolid dose that was completed by participants in each group. Table 3 presents the actual weeks of linezolid received in each group. The proportion of patients completing 26 weeks of linezolid at a dose of 1200 mg daily was 35 of 108 (32%) for the Nix-TB 1200-26 cohort and 31 of 44 (70%) in ZeNix 1200-26 cohort (overall, 66/152 [43%]). Among participants initially receiving 600 mg daily linezolid (ZeNix 600-26 and all arms of TB PRACTECAL), 320 of 354 (90%) participants were able to tolerate linezolid for the intended duration of at least 24 weeks. Five individuals who were unable to complete the intended duration of 600 mg daily linezolid, permanently ceased the drug or regimen (Table 4). For the remaining 29 participants, the daily dose of linezolid was reduced or interrupted, allowing completion of the intended regimen.

**Table 3.** Actual Weeks of Linezolid Received for Each BPaL-Containing Regimen

	Nix-TB Trial		ZeNix Trial								TB-PRACTECAL					
	BPaL 1200-26 Nix-TB		BPaL 1200-26 ZeNix		BPaL 1200-9 ZeNix		BPaL 600-26 ZeNix		BPaL 600-9 ZeNix		BPaL TB PRACTECAL		BPaLM TB PRACTECAL		BPaLC TB PRACTECAL	
Regimen	n (%)	Cumulative n (%)	n (%)	Cumulative n (%)	n (%)	Cumulative n (%)	n (%)	Cumulative n (%)	n (%)	Cumulative n (%)	n (%)	Cumulative n (%)	n (%)	Cumulative n (%)	n (%)	Cumulative n (%)
Total patients	n = 108		n = 44		n = 43		n = 43		n = 42		n = 102		n = 105		104	
Initial linezolid dose	1200 mg		1200 mg		600 mg		600 mg		600 mg		600 mg		600 mg		600 mg	
Target linezolid duration, wk	26		26		26		26		9		24^a^		24^a^		24^a^	
Weeks of linezolid																
0.0–2.9	2 (2%)	2 (2%)	1 (2%)	1 (2%)	1 (2%)	1 (2%)	1 (2%)	1 (2%)	1 (2%)	1 (2%)	2 (2%)	2 (2%)	5 (5%)	5 (5%)	3 (3%)	3 (3%)
3.0–5.9	15 (14%)	17 (16%)	0 (0%)	1 (0%)	4 (9%)	5 (12%)	0 (0%)	1 (2%)	1 (2%)	2 (5%)	4 (4%)	6 (6%)	3 (3%)	8 (8%)	3 (3%)	6 (6%)
6.0–8.9	10 (9%)	27 (25%)	4 (9%)	5 (11%)	35 (81%)	40 (93%)	1 (2%)	2 (5%)	38 (91%)	40 (84%)	2 (2%)	8 (8%)	0 (0%)	8 (10%)	2 (2%)	8 (8%)
9.0–11.9	19 (18%)	46 (43%)	3 (7%)	8 (18%)	3 (7%)	43(100%)	0 (0%)	2 (5%)	2 (5%)	42(100%)	1 (1%)	9 (9%)	3 (3%)	11 (10%)	2 (2%)	10 (10%)
12.0–14.9	16 (15%)	62 (57%)	2 (5%)	10 (23%)	na	na	0 (0%)	2 (5%)	na	na	6 (6%)	15 (15%)	2 (2%)	13 (11%)	2 (2%)	12 (12%)
15.0–17.9	7 (7%)	69 (64%)	2 (5%)	12 (27%)	na	na	2 (5%)	4 (9%)	na	na	87 (85%)	102 (100%)	92 (89%)	105 (100%)	92 (89%)	104 (100%)
18.0–20.9	14 (13%)	83 (77%)	2 (5%)	14 (32%)	na	na	0 (0%)	4 (10%)	na	na	na	na	na	na	na	na
21.0–23.9	2 (2%)	85 (79%)	25 (57%)	39 (89%)	na	na	37 (86%)	41 (95%)	na	na	na	na	na	na	na	na
≥24.0	23 (21%)	108 (100%)	5 (11%)	44 (100%)	na	na	2 (5%)	43 (100%)	na	na	na	na	na	na	na	na

Abbreviations: BPaL, bedaquiline, pretomanid, linezolid; BPaLC, bedaquiline, pretomanid, and linezolid with clofazimine; BPaLM, bedaquiline, pretomanid, linezolid with moxifloxacin; na, not applicable; TB, tuberculosis; TB PRACTECAL Pragmatic Clinical Trial for a more Effective, Concise and Less Toxic Regimen.

^a^In TB-PRACTECAL, the intended linezolid dose was 600 mg daily for 16 wk, followed by 300 mg daily for 8 wk.

**Table 4.** Comparison Between the Proportion of Individuals Experiencing 1 or More Adverse Events Resulting in Interruption/Discontinuation of Any Drug for BPaL-Containing Regimens

	ZeNix Trial								Nix-TB Trial		TB-PRACTECAL Trial					
	BPaL 600-9 ZeNix		BPaL 600-26 ZeNix		BPaL 1200-9 ZeNix		BPaL 1200-26 ZeNix		BPaL 1200-26 Nix-TB		BPaL TB PRACTECAL		BPaLM TB PRACTECAL		BPaLC TB PRACTECAL	
	n (%)	Risk Difference^d^ (95% CI)	n (%)	Risk Difference (95% CI)	n (%)	Risk Difference (95% CI)	n (%)	Risk Difference (95% CI)	n (%)	Risk Difference (95% CI)	n (%)	Risk Difference (95% CI)	n (%)	Risk Difference (95% CI)	n (%)	Risk Difference (95% CI)
Total	n = 42		n = 43		n = 43		n = 44		n = 108		n = 102		n = 105		n = 104	
Intended daily dose and duration of linezolid	600 mg daily, 9 wk		600 mg daily, 26 wk		1200 mg daily, 9 wk		1200 mg daily, 26 wk		1200 mg daily, 26 wk		600 mg daily, 24 wk^a^		600 mg daily, 24 wk^a^		600 mg daily, 24 wk^a^	
Any AE leading to discontinuation^b^	0 (0%)	Ref	1 (2%)	0.02 (−.06, .12)	0 (0%)	0.00 (−.08, .08)	4 (9%)^c^	0.09 (−.009, .21)	18 (17%)	0.17 (.06, .25)*	5 (5%)	0.05 (−.04, .11)	4 (4%)	0.04 (−.05, .09)	5 (5%)	0.05 (−.04, .11)
Adverse events of special interest																
QT prolongation leading to discontinuation	0 (0%)	Ref	0 (0%)	0.00 (−.08, .08)	0 (0%)	0.00 (−0.8, .08)	0 (0%)	0.00 (−.08, .08)	0 (0%)	0.00 (−.08, .03)	0 (0%)	0.00 (−.08, .04)	1 (1%)	0.01 (−.07, .05)	0 (0%)	0.00 (−.08, .04)
Peripheral neuropathy leading to discontinuation^e^	0 (0%)	Ref	0 (0%)	0.00 (−.08, .08)	0 (0%)	0.00 (−.08, .08)	2 (5%)^c^	0.05 (−.04, .15)	16 (15%)	0.15 (.06, .23)*	0 (0%)	0.00 (−.08, .04)	0 (0%)	0.00 (−.08, .04)	0 (0%)	0.00 (−.08, .04)
Optic neuritis leading to discontinuation	0 (0%)	Ref	0 (0%)	0.00 (−.08, .08)	0 (0%)	0.00 (−.08, .08)	2 (5%)	0.05 (−.04, .15)	1 (1%)	0.01 (−.08, .05)	0 (0%)	0.00 (−.08, .04)	0 (0%)	0.00 (−.08, .04)	0 (0%)	0.00 (−.08, .04)
Myelosuppression leading to discontinuation	0 (0%)	Ref	1 (2%)	0.02 (−.06, .12)	0 (0%)	0.00 (−.08, .08)	0 (0%)	0.00 (−.08, .08)	2 (2%)	0.02 (−.07, .07)	3 (3%)	0.03 (−.06, .08)	0 (0%)	0.00 (−.08, .04)	1 (1%)	0.01 (−.07, .05)
Hepatotoxicity leading to discontinuation	0 (0%)	Ref	0 (0%)	0.00 (−.08, .08)	0 (0%)	0.00 (−.08, .08)	0 (0%)	0.00 (−.08, .08)	0 (0%)	0.00 (−.08, .03)	1 (1%)	0.01 (−.07, .05)	2 (2%)	0.02 (−.07, .07)	2 (2%)	0.02 (−.07, .07)

^*^ *P* < 0.05.

Abbreviations: AE, adverse event; AESI, adverse events of special interest; BPaL, bedaquiline, pretomanid, linezolid; CI, confidence interval; Ref, reference; TB, tuberculosis; TB PRACTECAL, Pragmatic Clinical Trial for a more Effective, Concise and Less Toxic Regimen.

^a^In TB-PRACTECAL the intended linezolid dose was 600 mg daily for 16 wk and then 300 mg daily for 8 wk, or earlier if moderately tolerated.

^b^Includes AESI and any other adverse event.

^c^One person in ZeNix 1200-26 ceased the entire regimen (B, Pa, L); all others ceased linezolid only.

^d^Risk difference calculated using ZeNix 600-9 as the comparator.

^e^Peripheral neuropathy was analyzed in Nix-TB, ZeNix, and PRACTECAL trials by using Standard MedDRA Query (SMQ) of peripheral neuropathy (Nix-TB MedDRA version 22.1, ZeNix MedDRA version 23.0, TB-PRACTECAL MedDRA version 19.1 to 25. The SMQ of peripheral neuropathy consists of multiple preferred terms that could be attributed to peripheral neuropathy.

Among patients taking the Nix-TB 1200-26 regimen, discontinuation of linezolid due to an adverse event occurred in 18 of 108 (17%) patients. The most common cause for cessation of drug therapy was peripheral neuropathy, affecting 16 of 18 (89%) participants (Table 5). The ZeNix 1200-26 regimen was better tolerated, with only 1 individual discontinuing all 3 drugs due to adverse events, which resulted in a classification of treatment failure. Among other participants who discontinued drugs, only linezolid was ceased. Myelosuppression and hepatoxicity were also more frequent in the Nix-TB 1200-26 regimen compared with ZeNix 1200-26 regimen.

**Table 5.** Number of Participants Experiencing 1 or More Grade 3–4 Adverse Events of Special Interest, by Regimen

	ZeNix Trial								NiX-TB Trial		TB-PRACTECAL Trial							
Regimen	BPaL 600-9 ZeNix		BPaL 600-26 ZeNix		BPaL 1200-9 ZeNix		BPaL 1200-26 ZeNix		BPaL 1200-26 Nix-TB		BPaL TB-PRACTECAL		BPaLM TB-PRACTECAL		BPaLC TB-PRACTECAL		SoC TB-PRACTECAL	
	n (%)	Risk Difference^b^	n (%)	Risk Difference (95% CI)	n (%)	Risk Difference (95% CI)	n (%)	Risk Difference (95% CI)	n (%)	Risk Difference (95% CI)	n (%)	n (%)	Risk Difference (95% CI)	n (%)	Risk Difference (95% CI)	n (%)	Risk Difference (95% CI)	n (%)
Total patients	n = 42		n = 43		n = 43		n = 44		n = 108		n = 102		n = 105		n = 104		n = 108	
Intended daily dose and duration of linezolid	600 mg daily, 9 wk		600 mg daily, 26 wk		1200 mg daily, 9 wk		1200 mg daily, 26 wk		1200 mg daily, 26 wk		600 mg daily, 24 wk^a^		600 mg daily, 24 wk^a^		600 mg daily, 24 wk^a^		600 mg daily, 24 wk^a^	
QT prolongation grade 3–4	1 (2%)	Ref	0 (0%)	−0.02 (−.12, .06)	0 (0%)	−0.02 (−.12, .06)	0 (0%)	−.02 (−0.12, .06)	0 (0%)	−.02 (−0.12, .01)	0 (0%)	−0.02 (−.12, .01)	0 (0%)	−0.02 (−.12, .01)	2 (2%)	−0.01 (−.11, .05)	9 (8%)	0.06 (−.5, .13)
Peripheral neuropathy grade 3–4^c^	0 (0%)	Ref	0 (0%)	0.00 (−.08, .08)	0 (0%)	0.00 (−.08, .08)	0 (0%)	0.00 (−.08, .08)	24 (22%)	0.22 (.13, .31)*	0 (0%)	0.00 (−.08, .04)	0 (0%)	0.00 (−.08, .04)	0 (0%)	0.00 (−.08, .04)	1 (1%)	0.01 (−.08, .05)
Optic neuritis grade 3–4	0 (0%)	Ref	0 (0%)	0.00 (−.08, .08)	0 (0%)	0.00 (−.08, .08)	1 (2%)	0.02 (−.06, .12)	0 (0%)	0.00 (−.08, .03)	0 (0%)	0.00 (−.08, .04)	0 (0%)	0.00 (−.08, .04)	0 (0%)	0.00 (−.08, .04)	0 (0%)	0.00 (−.08, .03)
Myelosuppression grade 3–4	2 (5%)	Ref	0 (0%)	−0.05 (−.16, .04)	2 (5%)	−0.001 (−.12, .11)	0 (0%)	−0.05 (−.16, .04)	7 (7%)	0.02 (−.10, .09)	4 (4%)	−0.01 (−.12, .06)	7 (7%)	0.02 (−.10, .09)	5 (5%)	0.00 (−.11, .07)	12 (11%)	0.06 (−.06, .15)
Hepatotoxicity grade 3–4	3 (7%)	Ref	4 (9%)	0.02 (−.11, .16)	3 (7%)	−0.002 (−.13, .13)	5 (11%)	0.04 (−.09, .18)	14 (13%)	0.06 (−.07, .15)	4 (4%)	−0.03 (−.15, .04)	9 (9%)	0.01 (−.11, .10)	5 (5%)	−0.02 (−.15, .05)	12 (11%)	0.04 (−.09, .13)

^*^ *P* < 0.05.

Abbreviations: BPaL, bedaquiline, pretomanid, linezolid; BPaLC, bedaquiline, pretomanid, and linezolid with clofazimine; BPaLM, bedaquiline, pretomanid, linezolid with moxifloxacin; CI, confidence interval; Ref, reference; SoC standard of care; TB PRACTECAL Pragmatic Clinical Trial for a more Effective, Concise and Less Toxic Regimen.

^a^In TB-PRACTECAL, the intended linezolid dose was 600 mg daily for 16 wk and then 300 mg daily for 8 wk, or earlier if moderately tolerated.

^b^Risk difference calculated using ZeNix 600-9 as the comparator.

^c^Peripheral neuropathy was analyzed in Nix-TB, ZeNix, and PRACTECAL trials by using Standard MedDRA Query (SMQ) of peripheral neuropathy (Nix-TB MedDRA version 22.1, ZeNix MedDRA version 23.0, TB-PRACTECAL MedDRA version 19.1 to 25). The SMQ of peripheral neuropathy consists of multiple preferred terms that could be attributed to peripheral neuropathy.

The permanent discontinuation of linezolid was rare among individuals receiving either the 600-26, 600-9, or 1200-9 regimens (15/439, 3%). Two participants who permanently discontinued linezolid were assigned a treatment failure outcome (1 participant in the ZeNix 1200-26 group and 1 participant in the TB-PRACTECAL BPaLC group). The remaining participants who ceased linezolid while receiving a BPaL-containing regimen achieved a successful treatment outcome.

Although AESI were common (Supplementary Table 3), the vast majority were grade 1 or 2 (Supplementary Table 3). Peripheral neuropathy was more likely to be experienced among participants in South Africa (Supplementary Figure 1). A greater number of adverse events were noted for participants receiving high doses of linezolid for longer durations. Adverse events attributable to bedaquiline or pretomanid were frequently noted; however, most events were grade 1 or 2 and did not require treatment cessation.

### Comparative Analyses

Table 4 shows the proportions of patients experiencing adverse events resulting in treatment discontinuation, by linezolid dose. The proportions of treatment-related severe adverse events (grade 3–4) observed with each regimen are shown in Table 5. Grade 3–4 peripheral neuropathy was more frequent in those receiving the Nix-TB 1200-26 regimen than for those receiving the ZeNix 600-9 regimen (RD, .22; 95% CI, .13–.31). The proportion of adverse events reported by patients receiving lower doses and durations of linezolid was similar to those receiving the lowest dose of linezolid (ZeNix 600-9). The frequency of myelosuppressive events and peripheral neuropathy was lower in all ZeNix regimens compared with Nix-TB 1200-26 (Supplementary Table 4). Adverse events were more frequent in those receiving the TB PRACTECAL standard-of-care regimen than those receiving BPaL-containing regimens (Supplementary Table 5).

## DISCUSSION

Three recent clinical trials have evaluated the safety and effectiveness of 8 BPaL-containing regimens. The incidence of treatment-related grade 3 to 4 adverse events was highest among those taking regimens with an initial dose of 1200 mg daily linezolid. A starting dose of 600 mg per day of linezolid appeared to be the best tolerated. These studies have informed recent changes to WHO guidelines, which recommend the adoption of a regimen containing BPaLM for 6 months for the treatment of MDR/RR-TB without fluoroquinolone resistance and the BPaL regimen for 6–9 months for those with MDR/RR-TB and fluoroquinolone resistance [16] (the updated WHO definition for pre-XDR-TB) [3].

Severe adverse events were relatively uncommon among participants receiving each of the BPaL-containing regimens. Toxicity was lowest for those with a starting dose of 600 mg of linezolid. Nevertheless, lower-grade (grade 1 and 2) adverse events were frequently reported. These findings are consistent with previous studies in which bedaquiline and linezolid have been used—while adverse events of any grade are frequent [17], severe (grade 3–4) adverse events of any kind were less common [18, 19].

Peripheral neuropathy was among the most common adverse events leading to treatment discontinuation. At the highest dose and duration of linezolid, Nix-TB 1200 mg daily for 26 weeks, peripheral neuropathy required treatment cessation in 15% of patients. The reasons for this are multifactorial. Patients in all 3 studies were closely monitored for evidence of early neuropathy by regular clinical assessment during treatment, meaning that misclassification is unlikely. Fewer participants in the ZeNix 1200 mg regimen experienced adverse events. As the ZeNix trial was performed after the Nix-TB trial, the ability to adapt to early signs of peripheral neuropathy may have been more nuanced and may have prevented more severe events in the ZeNix trial. Interestingly, the incidence of peripheral neuropathy varied considerably by region, with the highest incidence among those treated in South Africa. Ultimately, this study demonstrates the importance of carefully monitoring patients for neuropathy throughout treatment with BPaL-containing regimens.

Optic neuropathy, another recognized complication of linezolid therapy, was infrequently observed in these 3 trials. In contrast, observational studies in other settings have measured the incidence of optic neuropathy, using monthly visual assessment, to be between 5.8% and 48.5% of participants taking a linezolid-containing regimen, although this was often reversible after cessation of linezolid [20, 21]. Myelosuppression, another commonly reported side effect of linezolid [20], was relatively uncommon with the BPaL-containing regimens, except in those receiving 1200 mg daily linezolid. While early phase 2 trials identified QTc prolongation as a potentially concerning adverse event attributable to bedaquiline, clofazimine, or moxifloxacin [22], this concern has not been borne out in subsequent cohort studies where treatment was given for a longer duration [23], or in the trials included in this review.

An important concern regarding the use of 3-drug regimens to treat MDR-TB is the potential for acquired drug resistance, particularly if one of the included antibiotics needs to be interrupted [24]. Reassuringly, few patients stopped treatment among those taking BPaL-containing regimens using 600 mg of linezolid. This needs to be confirmed in programmatic settings and, until then, including 4 drugs as per WHO guidelines may be an alternative. Permanent discontinuation of bedaquiline and pretomanid was also infrequent, consistent with previous studies, showing that toxicity-related cessation of bedaquiline is rare [25].

This study had several limitations. The number of participants in each treatment group was relatively small. Therefore, less common but serious complications of these therapies may not have been detected. Second, the monitoring for adverse events differed between the 3 studies, in particular for hepatotoxicity and myelosuppression. This may have contributed to differences in the frequency of lower grade events reported between studies—such as the higher incidence of grade1 and 2 events reported in the TB-PRACTECAL regimens. Third, a lack of standard-of-care arms in the Nix-TB and the ZeNix trials precluded a comparison of the toxicity with BPaL to established longer injectable-based or all oral regimens in these studies. In TB-PRACTECAL, a higher incidence of adverse events was noted in standard-of-care arms.

A key strength of the study was the inclusion of 3 recent trials, which allowed an unbiased comparison of the safety of different linezolid doses (ZeNix) and companion drugs (TB-PRACTECAL). The studies also monitored adverse events more closely compared with operational settings, permitting a broader understanding of the toxicity of BPaL-containing regimens. Furthermore, as the study included data provided in a public call, the risk of publication bias was reduced.

Further research is required to evaluate the tolerability of BPaL-containing regimens. As the new WHO guidelines are implemented more widely, routine monitoring for adverse events and for acquired drug resistance will be important. Furthermore, active TB Drug Safety Monitoring and Management will play an important role in guiding scale-up within national TB programs to detect less common adverse events [26]. There is some evidence that linezolid 300 mg daily has been used in MDR-TB successfully, although not as an initial dose in a shorter BPaL regimen [8, 27]. The optimal dose of linezolid may differ in individuals due to the pharmacokinetic properties of linezolid [28]. Therapeutic drug monitoring is 1 strategy that may balance the treatment toxicity and optimal dose of linezolid [29] and further study in this realm is required. While the BPaL-containing regimens have been shown to be effective when *M. tuberculosis* is susceptible to all 3 drugs, resistance to 1 or 2 drugs in the regimen may compromise the effectiveness of the regimen. Hence, the introduction of novel regimens must be accompanied by routine monitoring for drug resistance. New rapid molecular tools promise to expedite early detection of drug resistance [30].

In conclusion, BPaL-containing regimens offer a promising range of new options for patients with MDR/RR-TB and more advanced drug resistance. Three recent trials have demonstrated the safety and tolerability of these regimens in a clinical trial setting. In accordance with recent WHO recommendations, a starting dose of 600 mg linezolid daily appears to be the best tolerated, while remaining efficacious. The availability of more effective, better tolerated, and shorter treatment options for patients, such as BPaL-containing regimens, will make an important contribution to the global ambition to eliminate TB.

## Supplementary Data

Supplementary materials are available at *Clinical Infectious Diseases* online. Consisting of data provided by the authors to benefit the reader, the posted materials are not copyedited and are the sole responsibility of the authors, so questions or comments should be addressed to the corresponding author.

## Supplementary Material

ciad653_Supplementary_Data

## Notes

***Author Contributions.*** T. H.: conceptualization, data curation, formal analysis, methodology, writing—original draft; E. M.: data curation, formal analysis, writing—review and editing; B.-T. N.: data curation, writing—review and editing; E. E.: data curation, writing—review and editing; C. B.: data curation, writing—review and editing; M. D.: data curation, writing—review and editing; S. F.: data curation, writing—review and editing; M. G.: supervision, writing—review and editing; M. L.: data curation, writing—review and editing; F. M.: supervision, writing—review and editing; H. M.: data curation, writing—review and editing; I. M.: data curation, writing—review and editing; L. N.: supervision, writing—review and editing; S. S.: supervision, writing—review and editing; T. S.: supervision, writing—review and editing; G. F.: conceptualization, supervision, writing—review and editing.

***Acknowledgments.*** The authors acknowledge the contributions of the Nix, ZeNix and TB PRACTECAL trial teams for contributing their data to this meta-analysis. They thank the many patients participating in these trials.

***Financial support.*** This work was supported by funding provided by the World Health Organization. G. F. was supported by an Australian National Health and Medical Research Council Investigator Grant (APP2007920).
