# Supplementary material for: The Safety and Tolerability of Linezolid in Novel Short-Course Regimens Containing Bedaquiline, Pretomanid, and Linezolid to Treat Rifampicin-Resistant Tuberculosis: An Individual Patient Data Meta-analysis
Source: Clin Infect Dis. 2023 Oct 24;78(3):730–41. doi: 10.1093/cid/ciad653 (PMC10954324; doi:10.1093/cid/ciad653)
Supplement: ciad653_Supplementary_Data [file ciad653_supplementary_data.docx]

**Table S1: Adverse event related cut-offs by grade in clinical trials using BPaL-containing regimens**

|  | **Grade 1** | **Grade 2** | **Grade 3** | **Grade 4** |
| --- | --- | --- | --- | --- |
| **Nix-TB trial, ZeNix trial**  **(DMID, adult toxicity table, Nov 2007 draft)** | | | | |
| If no specific cut-off for specific event, here are the general definition | Transient or mild discomfort (<48 hours); no medical intervention/therapy required | Mild to moderate limitation in activity- some assistance may be needed; no or minimal medical intervention/therapy required. | Marked limitation in activity, some assistance usually required; medical intervention/therapy required, hospitalizations possible. | Extreme limitation in activity, significant assistance required; significant medical intervention/therapy required, hospitalization or hospice care probable |
| QT prolongation​ | < 450 msec | 450 msec ≤ QTcF < 480 msec OR  0 msec <change in QTcF ≤ 30 msec | 480 msec ≤ QTcF < 500 msec OR  30 msec <change in QTcF ≤60 msec | ≥ 500 msec OR  change in QTcF ≥ 60 msec |
| Peripheral neuropathy​ * |  |  |  |  |
| Paresthesia (burning, tingling, etc.) | Mild discomfort; no treatment required | Moderate discomfort; non-narcotic analgesia required | Severe discomfort; or narcotic analgesia required with symptomatic improvement | Incapacitating; or not responsive to narcotic analgesia |
| Neuro-Sensory | Mild impairment in sensation (decreased sensation, e.g. vibratory, pinprick, hot/cold in great toes) in focal area or symmetrical distribution; or change in taste, smell, vision and/or hearing | Moderate impairment (mod decreased sensation, e.g. vibratory, pinprick, hot/cold to ankles) and/or joint position or mild impairment that is not symmetrical | Severe impairment (decreased or loss of sensation to knees or wrists) or loss of sensation of at least mod degree in multiple different body areas (i.e., upper and lower extremities) | Sensory loss involves limbs and trunk; paralysis; or seizures |
| Myelosuppression​ |  |  |  |  |
| Haemoglobin | 9.5 - 10.5 gm/dL | 8.0 - 9.4gm/dL | 6.5 - 7.9 gm/dL | < 6.5 gm/dL |
| Platelets | 75,000-  99,999/mm3 | 50,000-  74,999/mm3 | 20,000-  49,999/mm3 | <20,000/mm3 |
| Absolute Neutrophil Count | 1000-1500/  mm3 | 750-999  /mm3 | 500-749/mm3 | <500  /mm3 |
| ALT/ AST​ /ALP | 1.1 - <2.0 x ULN | 2.0 – <3.0 x ULN | 3.0 – 8.0 x ULN | > 8 x ULN |
| Hyperbilirubinemia (when accompanied by any increase in other liver function test) | 1.1 - <1.25 x ULN | 1.25 - <1.5 x ULN | 1.5 - <1.75 x ULN | > 1.75 x ULN |
| Hyperbilirubinemia (when other liver function are in the normal range) | 1.1 - <1.5 x ULN | 1.5 - <2.0 x ULN | 2.0 – <3.0 x ULN | > 3.0 x ULN |
| ALT/ AST​ | 1.1 - <2.0 x ULN | 2.0 – <3.0 x ULN | 3.0 – 8.0 x ULN | > 8 x ULN |
| **TB-PRACTECAL** | | | | |
| QT prolongation​ (CTCAE 4.03) | Average QTcF 450 - 480 ms | Average QTcF 481 - 500 ms | Average QTcF >= 501 ms without signs/symptoms of serious arrhythmia | Average QTcF >= 501 or >60 ms change from baseline and one of the following: Torsade de pointes or polymorphic ventricular tachycardia or signs/symptoms of serious arrhythmia |
| Peripheral neuropathy​* |  |  |  |  |
| Neuro-Sensory Disorders (DMID) | Mild impairment in sensation (decreased sensation, e.g. vibratory, pinprick, hot/cold in great toes) in focal area or symmetrical distribution; or change in taste, smell, vision and/or hearing; and/or vibration | Moderate impairment (mod decreased sensation, e.g. vibratory, pinprick, hot/cold to ankles) and/or joint position; and/or vibration perception | Severe impairment (decreased or loss of sensation to knees or wrists); and/or vibration perception and/or deep tendon reflex | Sensory loss involves limbs and trunk; paralysis; or seizures; and/or deep tendon reflex. |
| Paresthesia (Burning, Tingling, etc.) (DMID) | Mild discomfort; no treatment required; | Moderate discomfort; non-narcotic analgesia required; | Severe discomfort; or narcotic analgesia required with symptomatic improvement | Incapacitating; or not responsive to narcotic analgesia. |
| Optic neuritis​ (Optic nerve disorder, CTCAE) | Asymptomatic; clinical or diagnostic observations only | Limiting vision of the affected eye (20/40 [6/12] or better) | Limiting vision in the affected eye (worse than 20/40 [6/12] but better than 20/200 [6/60]) | Blindness (20/200 [6/60] or worse) in the affected eye |
| Myelosuppression​ (DMID) |  |  |  |  |
| Absolute Neutrophil Count | 1500 - 1000/mm3 [1.5 - 1.0 x10^9/L] [1.5 - 1.0 x10^3/μL] | 999 - 750/mm3  [0.99 - 0.75 x10^9/L] [0.99 - 0.75 x10^3/μL] | 749 - 500/mm3  [0.74 - 0.50 x10^9/L] [0.74 - 0.50 x10^3/μL] | <500/mm3  [<0.50 x10^9/L] [<0.50 x10^3/μL] |
| Anaemia | 10.5 - 9.5 g/dL [105 - 95 g/L] | 9.4 - 8.0 g/dL  [94 - 80 g/L] | 7.9 - 6.5 g/dL  [79 - 65 g/L] | < 6.5 g/dL  [< 65 g/L] |
| Platelets | 99,999-75,000/mm3  [99.9-75.0 x10^9/L] [99.9-75.0 x10^3/μL] | 74,999-50,000/mm3  [74.9-50.0 x10^9/L] [74.9-50.0 x10^3/μL] | 49,999-20,000/mm3 [49.9-20.0 x10^9/L] [49.9-20.0 x10^3/μL] | <20,000/mm3  [<20.0 x10^9/L] [<20.0 x10^3/μL] |
| White Blood Cell | <LLN - 3000/mm3 [<LLN - 3 x10^9/L] [<LLN - 3 x10^3/μL] | <3000 - 2000/mm3 [3 - 2 x10^9/L] [3 - 2 x10^3/μL] | <2000 - 1000/mm3 [<2 - 1 x10^9/L] [<2 - 1 x10^3/μL] | <1000/mm3 [<1 x10^9/L] [<1 x10^3/μL] |
| Hepatotoxicity​ (CTCAE) |  |  |  |  |
| AST/ALT | >ULN - 3.0 x ULN | >3.0 - 5.0 x ULN | >5.0 - 20.0 x ULN | >20.0 x ULN |
| ALP | >ULN - 2.5 x ULN | >2.5 - 5.0 x ULN | >5.0 - 20.0 x ULN | >20.0 x ULN |
| Bil | Hyperbilirubinemia | >ULN - 1.5 x ULN | >1.5 - 3.0 x ULN | >3.0 - 10.0 x ULN |

ALP alakaline phosphatase, ALT alanine aminotransferase, AST asparate aminotransferase, CTCAE Common terminology Criteria for adverse events (US Department of Health and Human Services. *Common Terminology Criteria for Adverse Events (CTCAE)*, 2017), DMID Division of Microbiology and Infectious Diseases (National Institutes of Health (NIH) NI of A and ID (NIAID). Division of Microbiology and Infectious Diseases (DMID). Pediatric Toxicity Tables, November 2007 draft) QTcF corrected QTc using Fridericia´s formula, ULN upper limit of normal

Peripheral neuropathy was analysed in Nix-TB, ZeNix and PRACTECAL by using Standard MedDRA Query (SMQ) of peripheral neuropathy (Nix-TB MedDRA version 22.1, ZeNix MedDRA version 23.0, TB-PRACTECAL MedDRA version 19.1 to 25). The SMQ of peripheral neuropathy consists of multiple preferred terms that could be attributed to PN.

**Table S2: End of treatment outcomes according to WHO definitions, grouped by regimen**

|  | **Nix-TB trial** | **ZeNix trial** | | | | **TB-PRACTECAL** | | | |
| --- | --- | --- | --- | --- | --- | --- | --- | --- | --- |
| **Clinical characteristics** | **BPaL *1200-26 Nix-TB*** | **BPaL *1200-26 ZeNix*** | **BPaL 1200-9 ZeNix** | **BPaL  600-26 ZeNix** | **BPaL 600-9 ZeNix** | **BPaL TB PRACTECAL** | **BPaLM TB PRACTECAL** | **BPaLC TB PRACTECAL** | **SoC TB PRACTECAL** |
|  | **n (%)** | **n (%)** | **n (%)** | **n (%)** | **n (%)** | **n (%)** | **n (%)** | **n (%)** | **n (%)** |
| **Total** | **108** | **44** | **43** | **43** | **42** | **105** | **104** | **102** | **108** |
| **Successful outcome** | 102 (94%) | 43 (98%) | 41 (95%) | 43 (100%) | 40 (95%) | 94 (90%) | 87 (84%) | 83 (81%) | 63 (58%) |
| **Lost to follow up** | 0 (0%) | 0 (0%) | 0 (0%) | 0 (0%) | 1 (2%) | 5 (5%) | 8 (8%) | 10 (10%) | 21 (19%) |
| **Failure** | 0 (0%) | 1 (2%) | 1 (2%) | 0 (0%) | 1 (2%) | 6 (6%) | 8 (8%) | 8 (8%) | 21 (19%) |
| **Death** | 6 (6%) | 0 (0%) | 1 (2%) | 0 (0%) | 0 (0%) | 0 (0%) | 1 (1%) | 1 (1%) | 3 (3%) |

DR-TB drug resistant tuberculosis, SoC standard of care, TB tuberculosis, TBP TB PRACTECAL

**Table S3: Number of participants experiencing one or more adverse events of special interest (any Grade) in BPaL containing regimens**

|  | **Nix-TB trial** | **ZeNix trial** | | | | **TB-PRACTECAL** | | | |
| --- | --- | --- | --- | --- | --- | --- | --- | --- | --- |
| **​** | **BPaL *1200-26 Nix-TB*** | **BPaL *1200-26 ZeNix*** | **BPaL 1200-9 ZeNix** | **BPaL  600-26 ZeNix** | **BPaL 600-9 ZeNix** | **BPaL TB-PRACTECAL** | **BPaLM TB-PRACTECAL** | **BPaLC TB-PRACTECAL** | **SoC TB-PRACTECAL** |
|  | **n (%)** | **n (%)** | **n (%)** | **n (%)** | **n (%)** | **n (%)** | **n (%)** | **n (%)** | **n (%)** |
| **Total patients​** | ***N=108***​ | ***N=44***​ | **n=43**​ | **n=43**​ | **n=42**​ | **n=102**​ | **n=105**​ | **n=104**​ | **n=108**​ |
| Intended daily dose of linezolid​ | 1200mg daily | 1200mg daily | 1200mg daily | 600mg daily | 600mg daily | 600mg daily | 600mg daily | 600mg daily | 600mg daily |
| **AEs of special interest* (any Grade)​** | **104 (96%)**​ | **28 (64%)**​ | **19 (44%)**​ | **14 (33%)**​ | **16 (38%)**​ | **82 (80%)**​ | **92 (88%)**​ | **91 (88%)**​ | **103 (95%)**​ |
| **QTc prolongation​** | 6 (6%)​ | 0 (0%)​ | 1 (2%)​ | 0 (0%)​ | 1 (2%)​ | 26 (25%)​ | 36 (34%)​ | 43 (41%)​ | 68 (63%)​ |
| **Peripheral neuropathy​*** | 85 (79%)​ | 12 (27%)​ | 7 (16%)​ | 4 (9%)​ | 3 (7%)​ | 7 (7%)​ | 4 (4%)​ | 11 (11%)​ | 22 (20%)​ |
| **Optic neuritis** | 1 (1%)​ | 4 (9%)​ | 0 (0%)​ | 0 (0%)​ | 0 (0%)​ | 1 (1%)​ | 3 (3%)​ | 2 (2%)​ | 2 (2%)​ |
| **Myelosuppression​** | 43 (40%)​ | 11 (25%)​ | 6 (14%)​ | 4 (9%)​ | 6 (14%)​ | 43 (42%)​ | 54 (51%)​ | 49 (47%)​ | 66 (61%)​ |
| **Hepatotoxicity​** | 31 (29%)​ | 11 (25%)​ | 9 (21%)​ | 9 (21%)​ | 8 (19%)​ | 60 (59%)​ | 57 (54%)​ | 65 (63%)​ | 69 (64%)​ |

 BPaL bedaquiline, pretomanid, linezolid, Lzd Linezolid, SoC standard of care, TB tuberculosis

*Peripheral neuropathy was analysed in Nix-TB, ZeNix and PRACTECAL by using Standard MedDRA Query (SMQ) of peripheral neuropathy (Nix-TB MedDRA version 22.1, ZeNix MedDRA version 23.0, TB-PRACTECAL MedDRA version 19.1 to 25). The SMQ of peripheral neuropathy consists of multiple preferred terms that could be attributed to PN.

**Table S4:** **Number of participants experiencing one or more Grade 3-4 adverse events in regimens containing BPaL using Nix-TB 1200mg-26 as a comparator**

|  | **Nix-TB trial** |  | **ZeNix trial** | | | | | | | |
| --- | --- | --- | --- | --- | --- | --- | --- | --- | --- | --- |
| ​ **Regimen** | **BPaL *1200-26 Nix-TB^*** |  | **BPaL *1200-26 ZeNix*** |  | **BPaL 1200-9 ZeNix** |  | **BPaL  600-26 ZeNix** |  | **BPaL 600-9 ZeNix** |  |
|  | **n(%)** | **Risk Difference (95% CI)** | **n(%)** | **Risk Difference (95% CI)** | **n(%)** | **Risk Difference (95% CI)** | **n(%)** | **Risk Difference (95% CI)** | **n(%)** | **Risk Difference (95% CI)** |
| **Total patients**​ | ***N=108***​ |  | ***N=44***​ |  | **n=43**​ |  | **n=43**​ |  | **n=42**​ |  |
| Intended daily dose of linezolid​ | 1200mg daily |  | 1200mg daily |  | 1200mg daily |  | 600mg daily |  | 600mg daily |  |
| QT prolongation​ | 0 (0%)​ | Ref | 0 (0%)​ | ND | 0 (0%)​ | ND | 0 (0%)​ | ND | 1 (2%)​ | 0.02 (-0.02, 0.07) |
| Peripheral neuropathy​** | 24 (22%)​ | Ref | 0 (0%)​ | **-0.22 (-0.30, -0.14)** | 0 (0%)​ | **-0.22 (-0.30, -0.14)** | 0 (0%)​ | **-0.22 (-0.30, -0.14)** | 0 (0%)​ | **-0.22 (-0.30, -0.14)** |
| Optic neuritis | 0 (0%)​ | Ref | 1 (2%)​ | 0.02 (-0.02, 0.07) | 0 (0%)​ | ND | 0 (0%)​ | ND | 0 (0%)​ | ND |
| Myelosuppression​ | 7 (7%)​ | Ref | 0 (0%)​ | **-0.06 (-0.11, -0.02)** | 2 (5%)​ | -0.02 (-0.10, 0.06) | 0 (0%)​ | **-0.06 (-0.11, 0.02)** | 2 (5%)​ | -0.02 (-0.10, 0.06) |
| Hepatotoxicity​ | 14 (13%)​ | Ref | 5 (11%)​ | -0.02 (-0.13, 0.10) | 3 (7%)​ | -0.06 (-0.16, 0.04) | 4 (9%)​ | -0.04 (-0.14, 0.07) | 3 (7%)​ | -0.06 (-0.16, 0.04) |

ND no difference

*Bolded text indicates p<0.05

**Peripheral neuropathy was analysed in Nix-TB, ZeNix and PRACTECAL by using Standard MedDRA Query (SMQ) of peripheral neuropathy (Nix-TB MedDRA version 22.1, ZeNix MedDRA version 23.0, TB-PRACTECAL MedDRA version 19.1 to 25). The SMQ of peripheral neuropathy consists of multiple preferred terms that could be attributed to PN.

**Table S5: Number of participants experiencing one or more Grade 3-4 adverse events in regimens containing BPaL using TB PRACTECAL standard of care as a comparator**

| ​ **Regimen** | **SoC TB-PRACTECAL^** | | **BPaL TB-PRACTECAL** | | **BPaLM TB-PRACTECAL** | | **BPaLC TB-PRACTECAL** | |
| --- | --- | --- | --- | --- | --- | --- | --- | --- |
|  | **n(%)** | **Risk Difference (95% CI)** | **n(%)** | **Risk Difference (95% CI)** | **n(%)** | **Risk Difference (95% CI)** | **n(%)** | **Risk Difference (95% CI)** |
| **Total patients**​ | **n=108**​ |  | **n=102**​ |  | **n=105**​ |  | **n=104**​ |  |
| Intended daily dose of linezolid​ | 600mg daily |  | 600mg daily |  | 600mg daily |  | 600mg daily |  |
| QT prolongation​ | 9 (8%)​ | Ref | 0 (0%)​ | **-0.08 (-0.15, -0.04)** | 0 (0%)​ | **-0.08 (-0.15, -0.04)** | 2 (2%)​ | **-0.06 (-0.13, –0.01)** |
| Peripheral neuropathy*​ | 1 (1%)​ | Ref | 0 (0%)​ | -0.01 (-0.05, 0.03) | 0 (0%)​ | -0.01 (-0.05, 0.03) | 0 (0%)​ | -0.01 (-0.05, 0.03) |
| Optic neuritis​ | 0 (0%)​ | Ref | 0 (0%)​ | -0.00 (-0.03, 0.04) | 0 (0%)​ | -0.00 (-0.03, 0.04) | 0 (0%)​ | -0.00 (-0.03, 0.04) |
| Myelosuppression​ | 12 (11%)​ | Ref | 4 (4%)​ | **-0.07 (-0.15, -0.0001)** | 7 (7%)​ | -0.04 (-0.13, 0.03) | 5 (5%)​ | -0.06 (-0.14, 0.01) |
| Hepatotoxicity​ | 12 (11%)​ | Ref | 4 (4%)​ | **-0.07 (-0.15, -0.0001)** | 9 (9%)​ | -0.03 (-0.11, 0.06) | 5 (5%)​ | -0.06 (-0.14, 0.01) |

^ reference for comparative analysis

*Peripheral neuropathy was analysed in Nix-TB, ZeNix and PRACTECAL by using Standard MedDRA Query (SMQ) of peripheral neuropathy (Nix-TB MedDRA version 22.1, ZeNix MedDRA version 23.0, TB-PRACTECAL MedDRA version 19.1 to 25). The SMQ of peripheral neuropathy consists of multiple preferred terms that could be attributed to PN.

**Figure S1: Proportion of participants experiencing an adverse event (any grade), attributable to peripheral neuropathy* by country of origin (* by SMQ of peripheral neuropathy):** **number of events/total population.**
